# UV-Mediated Photofunctionalization of Dental Implant: A Seven-Year Results of a Prospective Study

**DOI:** 10.3390/jcm9092733

**Published:** 2020-08-24

**Authors:** Makoto Hirota, Tomomichi Ozawa, Toshinori Iwai, Kenji Mitsudo, Takahiro Ogawa

**Affiliations:** 1 Weintraub Center for Reconstructive Biotechnology, Division of Advanced Prothodontics, UCLA School of Dentistry, Los Angeles, CA 90095-1668, USA; togawa@dentistry.ucla.edu; 2 Department of Oral and Maxillofacial Surgery/Orthodontics, Yokohama City University Medical Center, Yokohama, Kanagawa 232-0024, Japan; 3 Department of Oral and Maxillofacial Surgery, Yokohama City University Graduate School of Medicine, Yokohama, Kanagawa 236-0004, Japan; ozawa@yokohama-cu.ac.jp (T.O.); iwai104oams@yahoo.co.jp (T.I.); mitsudo@yokohama-cu.ac.jp (K.M.)

**Keywords:** photofunctionalization, dental implant, implant stability, osseointegration, oral cancer

## Abstract

Our objective was to evaluate the seven-year results of photofunctionalized implants placed in regular, complex, and cancer-related cases. This study was a prospective, single-center study. Photofunctionalization was performed immediately prior to implantation with Ultraviolet (UV) light for 15 minutes. The success rate of each patient group and the influential factors on implant failure were analyzed. Seventy implants in 16 patients were included. Four implants were left submerged (sleep). The seven-year success rate of 30 implants in regular cases and 21 implants in complex cases was 100%. The success rate of 15 implants in cancer-related cases was 22.2%, in which implants were placed in resection or reconstructed sites with or without pre- or postoperative radiation history. Implant stability quotient (ISQ) values increased at second-stage surgery by 3.2 in regular cases and by 21.9 in complex cases, while it decreased by −3.5 in cancer cases. Multivariate analysis indicated that bone quality, location, and cancer resection significantly influenced implant failure. A very reliable seven-year success rate was obtained by UV-photofunctionalized implants in regular and complex cases, even with significant site-development procedures. However, the success rate in cancer cases was significantly and remarkably lower, suggesting remaining challenges of pathophysiologically compromised conditions, such as bone resection, segmental defect, and radiation.

## 1. Introduction

Biological aging of titanium is a spontaneous and unavoidable phenomenon that starts immediately after the product is manufactured [1]. Both the smooth machine surface and acid etched surface are affected by biological aging in the same manner, without exception. Moreover, the process of aging is completed at just four weeks after production [2,3]. Aged titanium surfaces are covered with hydrocarbon, although the original surface should be titanium dioxide [3]. Over 50% of carbon has been detected on aged titanium surfaces, while the rate of titanium detection on the surface is only less than 20% on X-ray photoelectron spectroscopy (XPS) analysis [4]. Depending on the amount of accumulated carbon, osteoblast attachment, cell proliferation, and calcification behavior on the surface are compromised, resulting in approximately 60% of bone-to-implant contact (BIC), even though the BIC exceeds 90% when the titanium surface is fresh [3,4]. This suggests that hydrocarbon accumulation on titanium surfaces possibly impairs osseointegration. However, 60% of BIC is commonly accepted in current dental implantology, using commercially available as-made titanium [5,6,7]. Higher BIC values enable titanium to more strongly connect with bone. The mechanical strength of connection between bone and fresh titanium surface is three times greater than that of aged titanium surfaces [4].

UV-mediated photofunctionalization has been proposed to negate the biological aging of titanium, enhancing its adhesive property, achieving mostly 100% BIC, and increasing osseointegration strength [4,8]. This technique has been achieved after just 15 min, by using specific-wavelength UV light, to remove the hydrocarbon accumulated on titanium surfaces.

It has been reported that photofunctionalization could shorten the healing period without lowering the success rate [9], establish fast and rigid secondary osseointegration without impairing stability (so-called “stability-dip”) during the healing process [10], and develop implant stability even though the initial stability was extremely low by performing simultaneous bone augmentation [11].

These findings of photofunctionalization raise hopes of advancing indications for dental implant therapy in anatomically or pathophysiologically compromised bone. We hypothesized that photofunctionalization can successfully establish secure osseointegration, even though the implant is placed into the severely compromised bone. Severely atrophied jaws, which are considered to be anatomically compromised sites, are frequently encountered, and a staged-approach to dental implant therapy is often indicated, to obtain secure implant stability; however, the survival rate of these implants is still low compared to regular placement [12,13,14,15].

Dental implant therapy for oral cancer survivors seems to be more difficult than in regular cases [16]. Difficulties depend on prior treatment, such as surgery and/or radiation. Dental implant therapy in patients who have undergone tumor ablation could be a powerful tool to restore occlusion [16]; however, the jaw that has been anatomically and pathophysiologically compromised could be at a disadvantage with respect to establishing stability of the dental implant. In other instances, jaw bones could be pathophysiologically compromised by radiation therapy against oral cancer. Particularly, implant placement is contraindicated in patients who have received radical doses of radiation [17,18]. Implant surgery has been associated with a significantly increased risk of hard and soft tissue necrosis in the irradiated site [19]. These anatomical and pathophysiological problems could be a concern when considering indications for dental implant placement in oral cancer survivors.

The aim of this prospective study was to investigate the seven-year success rate of photofunctionalized implant placement in patients with regular, complex, and cancer-related cases.

## 2. Materials and Methods

### 2.1. Study Design

This study was a prospective study that evaluated the 7-year results of photofunctionalized implants, which were used in patients who underwent implant therapy at Yokohama City University Hospital, from 2011 to 2012. The same surgeon who was a nationally certified specialist in oral and maxillofacial surgery performed all the procedures. Inclusion criteria were ≥20 years of age, signed written informed consent, and at least one missing tooth in the jaws. Exclusion criteria were ongoing treatment of other oral diseases, the administration of over 60 Gy radiotherapy to the missing tooth area, taking bisphosphonate or denosumab, severe asthma or allergy, and pregnancy. The patients’ past history that could have affected the anatomical, physiological, or pathological bone condition of their maxilla or mandible was assessed. Bone quality and quantity based on computed tomography (CT) data were also assessed. Bone quality was classified as follows: D1 (hard bone), >1250 Hounsfield unit (HU); D2, 750–1250 HU; D3, 375–750 HU; D4, 150–375 HU; and D5 (soft or immature bone), <150 HU. Bone quantity was classified as follows: A, most of the alveolar bone is present; B, moderate ridge resorption; C, advanced residual resorption; D, moderate resorption of the basal bone; and E, extreme resorption of the basal bone. Yokohama City University Hospital ethics committee approved the study protocol (B110707028). 

### 2.2. Surgical Procedure and Implant Stability Quotient (ISQ) Measurement

All implants were loaded by using a staged approach. The healing period of implants after placement was determined according to placement procedures performed in regular and complex cases. A regular case was defined as implant placement into a normal site where bone augmentation was not needed to place the implant that had a regular platform, with a length of at least 8.5 mm. A complex case was defined as implant placement into a compromised site, such as an atrophied jaw, in which regular placement of a regular platform implant with at least 8.5 mm in length was difficult. Cases with simultaneous bone augmentation and implant placement into the tooth extraction socket were also deemed as complex cases. Then, oral-cancer-related cases, in which implants were placed into resected and/or irradiated bone, were distinctly sub-grouped. The healing period in a regular case was two months for the mandible and three months for the maxilla. The healing period in a complex case was six to eight months, regardless of location in the maxilla or mandible. The follow-up period was seven years after final prosthesis. Bone condition was accessed by annual radiographic examination. The implant systems used in this study were Brånemark System^®^ Mark III Groovy (67 implants) and NobelReplace^®^ Tapered Groovy (3 implants) (Nobel Biocare, Gothenburg, Sweden). Implant stability was measured by using resonance frequency analysis (RFA) with Ostell ISQ (Ostell, Gothenburg, Sweden) at both implant placement (ISQ1) and secondary surgery (ISQ2). Success of implants was assessed according to Albreksson’s criteria [20]. The overall success rate, success rate in regular, complex, and cancer-related cases were analyzed.

### 2.3. Photofunctionalization and Implant-Placement Procedure

**Figure 1:**
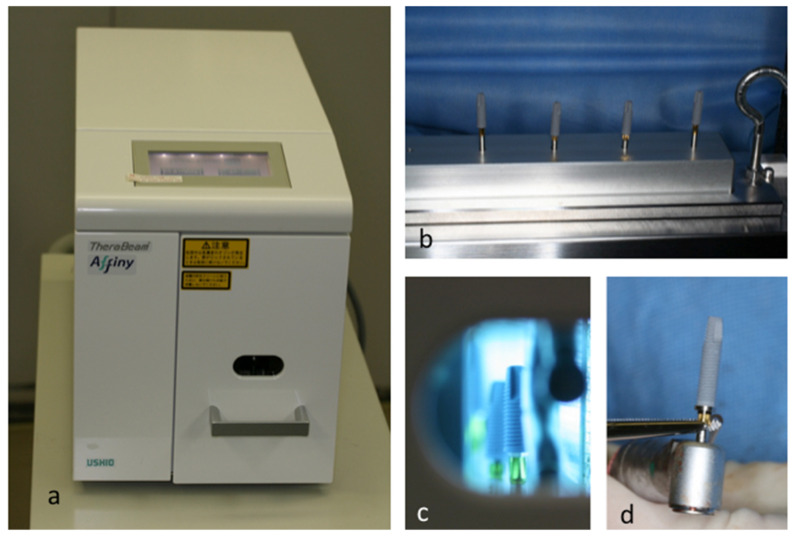
Device for photofunctionalization on dental implants: (**a**) TheraBeam^®^ Affiny and (**b**) implant stand. Six implants can be set. (**c**) Dental implant is treated with Ultraviolet light, (**d**) How to set a photofunctionalized implant into a handpiece.

Photofunctionalization was performed immediately prior to implantation by treating the implants with UV light for 15 min, at chairside, using a photo device (TheraBeam Affiny, Ushio, Tokyo, Japan) (Figure 1a). The dental implant was set on the stand table, through an implant driver (Figure 1b), the table was placed into the chamber of the device, and the button to start UV-irradiation was pressed. After 15 min, UV treatment was followed by a 5 min treatment to clean ozone (Figure 1c), and photofunctionalization was completed. The chamber was opened, and the photofunctionalized implant picked up carefully. Then the implant was repositioned to the handpiece head with straight Pean forceps (Figure 1d). After setting the photofunctionalized implant in the handpiece, the dentist carefully placed the implant into the implantation socket, without touching any other fluid, device, and tissue.

### 2.4. Statistical Analyses

The success rate was calculated after subtracting the number of withdrawn implants from the number of remaining implants in each study year. Multivariate regression analysis was performed with statistical software (StatFlex, Osaka, Japan), to detect the influence of patient age and sex, implant site, and diameter and length of implants on implant failure in both regular and complex cases. The difference was considered as significant when the *p*-value was less than 0.05.

## 3. Results

A total of 70 implants in 16 patients were included in this study. The average age of patients was 56.8 (range, 34–70) years. Four were female, and 12 were male. Case description is summarized in Table 1. Two patients had diabetes, three patients were smokers, and three were oral cancer survivors who had undergone marginal or segmental mandibular resection. One patient had undergone the marginal mandibular resection against recurrent cancer after radiation therapy. One patient had undergone segmental mandibular resection after preoperative radiation therapy and immediate reconstruction using fibular free flap. The other patient had undergone the marginal mandibular resection and bone augmentation with particulate cancellous bone and marrow (PCBM), which was harvested from the iliac crest on cancer resection site before implant insertion. One to fourteen implants were placed in each patient, to support a single crown, fixed partial, or complete denture. 

**Figure 2:**
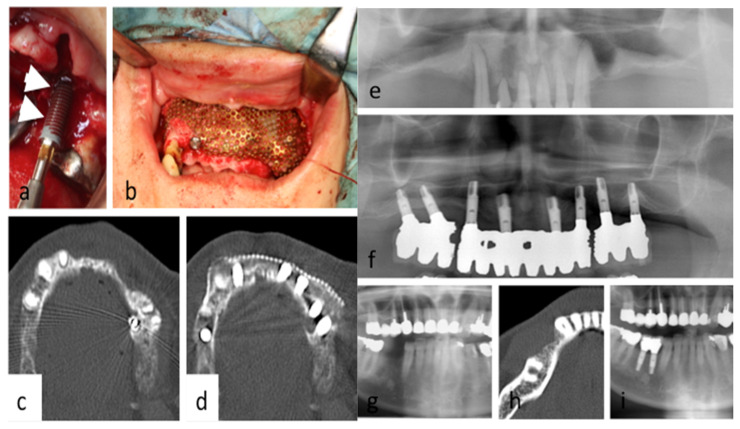
(**a**) Photofunctionalized implant attracts blood. (**b**) Simultaneous bone augmentation with PCBM and titanium mesh in atrophied maxilla. Computed tomography before (**c**) and after (**d**) placement with simultaneous bone augmentation (**a**–**d**; Pt.No.7). Orthopantomography before implantation (**e**) and after prosthesis (**f**) (Pt.No.4). Orthopantomography (**g**) and computed tomography (CT) (**h**) before implantation. Orthopantomography (**i**) five years after final prosthesis (**g**–**i**; Pt.No.2).

The implant and bone profiles, such as implant length and diameter and bone quantity and quality, are summarized in Table 2. Among the 70 implant sites, 34 were placed in regular sites, 21 were placed in complex sites, and 15 were placed in cancer-related sites. Photofunctionalized implants attracted blood (Figure 2a). Four implants in regular sites did not progress to secondary surgery, because the treatment plan was changed (sleep). Besides the regular placement, 21 implants were placed in complex sites, which were anatomically compromised, like atrophied jaw, and 15 were placed in cancer-related sites, which were pathophysiologically compromised because of cancer therapy, such as resection and radiation. Twenty-one implants in complex sites were placed with simultaneous/staged bone augmentation or immediately after tooth extraction: eight were placed with simultaneous and two with staged sinus lift, using PCBM harvested from the tibia or iliac crest; six with simultaneous guided bone regeneration (GBR), using Ti mesh and PCBM harvested from the tibia or iliac crest (Figure 2b–d); three in fresh extraction socket; and two with GBR, using absorbable membrane and chin bone (Figure 2e–g). In cancer-related sites, seven were placed into irradiated sites, followed by bone reconstruction using fibular osteocutaneous flap; four were placed into bone resection sites followed by bone augmentation, using Ti mesh and iliac PCBM; and four were placed into resected and irradiated bone. Three patients were withdrawn on the third to fourth year of the study period because they required cancer treatment. Of the 34 implants in regular cases, two implants were withdrawn, and of the 15 implants in cancer-related sites, one failed before final prosthesis and two failed three-and-a-half years after final prosthesis, and six implants were withdrawn.

The cumulative overall success rates according to survival implants are shown in Table 3. The success rate was over 90% up to the third year of the observation period. Eight implants were withdrawn at three−four years of the follow-up period because the patients needed to undergo treatment for other diseases. The overall seven-year success rate of implants was 87.9%, and the success rates of implants in regular and complex cases were 100%. The success rate of implants placed into cancer-related sites was 22.2% (see Table 4). The ISQ 1 of overall and in regular, complex, and cancer-related sites was 52.6 and 67.2, 30.5, and 62.1, respectively. The ISQ 2 of overall and in regular, complex, and cancer-related sites was 66.3 and 70.4, 52.4, and 58.6, respectively. ISQ increase or decrease in overall and in regular, complex, and cancer-related sites was 13.7 and 3.2, 21.9, and −3.5, respectively (see Table 5). Multivariate analysis indicated that bone quality and location (implant site) significantly influenced implant failure. Surgical resection of the jaw was the main factor that influenced implants’ success. Hard (D1) or soft (D5) bone and placed in the mandible and posterior site were significant risk factors for implant failure (*p* < 0.05). Then, radiation therapy considerably and possibly influenced implant failure, although it was not statistically significant (*p* = 0.092) (see Table 6).

## 4. Discussion

The present study was the first prospective study of seven-year results of photofunctionalized implants. The overall seven-year success rate of photofunctionalized implant was 87.9%, which is equivalent or relatively lower than previous studies [21], even though the success rate in regular and complex cases was 100%. The reason for the low overall success rate was considered that the success rate of cancer-related sites was remarkably low, suggesting that cancer-related treatment to the jaws contributed to the reduced success rate.

The present study included a few oral cancer survivors. Fifteen implants were placed into oral cancer treatment sites (resected or resected and irradiated site), but only two implants successfully survived for five years. Dental implant therapy for oral cancer survivors seems to be more difficult compared to regular cases [16]. Particularly, implant placement is contraindicated in patients who have received radical doses of radiation [17,18]. In the present study, four implants were placed into irradiated bone, but two of them resulted in late failure because of peri-implantitis. In addition, reconstruction of the tumor ablative region was also characteristic when a dental implant was indicated in oral cancer survivors [22]. Seven implants were placed into fibular bone flap, and four were placed into the bone previously reconstructed with PCBM. However, the implant into the fibular bone flap failed to integrate, and all implants into the augmented bone with PCBM caused peri-implantitis. A common point of these failures in irradiated and reconstructed cases was that the implants were placed into the bone with poor quality. Two failed and one non-integrated implants were placed into hard bone (bone quality D1), and four failed implants were placed into soft bone (bone quality D5). In other words, photofunctionalized implants placed into the bone, which was pathophysiologically compromised because of cancer-related treatment, could not establish long-term stability, suggesting photofunctionalization could never be an omnipotent technique that can result in dental implant success in any bone quality. Some cases in the present study were unable to continue follow-up for seven years because of other diseases, and this could be a reason for the relatively low success rate. Their dental implants were successfully observed during the follow-up period, indicating they would have been deemed a “success” had they completed the required follow-up. Dole et al. [23] reported that 3-, 7-, and 11-year implant survival rate in oral cancer patients was 94.9%, 92.5%, and 90.8%, respectively. They also reported that tumor ablative surgery followed by radiochemotherapy significantly reduced implant survival rate. The present result of cancer-patients-related implant was remarkably low compared to their study. However, in terms of “survival rate” (not “success rate”), our result of “survival rate” was 100% at four years, even in cancer-related cases. Linsen et al. [24] also reported that 5- and 10-year implant survival rate following radical oral cancer was 96.6% and 86.9%. Both reports [23,24] gradually reduced the survival rate around the 10-year follow-up period, in common, suggesting difficulties of management of dental implant after oral cancer treatment. However, a recent study of implant for non-oral-cancer cases but other regular and complex cases showed a high long-term success rate of 96.8% at five years [25] and 92.3% at 10 years [26]. Then, Cotic et al. [27] reported bone transplantation after oral cancer surgery was a significant risk factor for implant failure, indicating only reconstructing bone defects was still far from enough for success of dental implant in oral cancer patients. A lack of keratinized gingiva, saliva, and neuromuscular system are thought to reduce success and survival rate of dental implants. Therefore, careful attention on poor bone quality should be paid even though the bone quantity was sufficient in cancer-related cases.

Unlike photofunctionalized implants placed into cancer treatment sites, the implants placed into atrophied bone were successful when the bone condition of the residual bone was healthy (D2–4). Considering that the success rate of dental implants placed with bone augmentation was generally low compared to regular implant placement [9], we see that the present result might provide the motivation for the strong advancement of this procedure, suggesting that photofunctionalization can expand the indications for simultaneous implant placement with bone augmentation, even in severely anatomically compromised bone. The essence of photofunctionalization is to clean the titanium surfaces, which are spontaneously contaminated with natural hydrocarbon over time, to optimize the ability to establish osseointegration, regardless of the surface property. The carbon accumulation on aged titanium surface reduces up to less than 20%, and the original form of titanium dioxide is exposed [4]. Osteoblast attachment on photofunctionalized titanium surface remarkably increases, and consequently, rigid bone integration has been achieved with mostly 100% BIC [4]. Clinically, we previously reported that even with initial bone support of less than 25% of implant length or ISQ of less than 30, photofunctionalization can achieve secure secondary stability [11], indicating that photofunctionalization can overcome extremely low initial stability and/or less bone support. The reason why photofunctionalized implants achieve secure secondary stability even when the initial stability is quite low is that photofunctionalized implant achieves faster and higher osseointegration compared to as-received implants [10]. Photofunctionalization negates temporal decreases in implant stability during the healing process in what is known as the “stability dip” [10]. The key essence of osseointegration on photofunctionalized titanium surface is its superhydrophilicity, its being carbon free, and its positively charged electrical status [8]. Superhydrophilic surfaces can effectively attract blood to support the achievement of osseointegration [11], and a positively electrically charged surface allows osteoblasts to rigidly attach to the surface, because osteoblasts are negatively charged [28]. Then, photofunctionalization significantly reduces bacterial contamination and biofilm formation on aged, as-made titanium surface [29,30]. Not surprisingly, bacterial contamination on titanium surfaces is an unfavorable event when trying to avoid peri-implantitis. However, hydrophobicity is a main driving force for bacterial adhesion [31]. Therefore, converting hydrophobic to superhydrophilic surfaces by photofunctionalization has an antibacterial effect. Then, photofunctionalization could restore osseointegration in patients with type 2 diabetes [32].

In addition, photofunctionalization reduces the production of reactive oxygen species (ROS), which could induce cell apoptosis [33], resulting in enhancement of osteoblastic behavior attached on titanium surfaces. The advantageous bone healing on photofunctionalized titanium implant was also revealed by using a gap-healing model-assumed insertion for the extraction socket [34]. In that model, photofunctionalized implants effectively attracted new bone formation into the gap, indicating that the titanium surface can recruit and retain more osteogenic cells for direct bone formation at the implant surface in what we call “contact osteogenesis” [35]. In the augmentation model using titanium mesh, the exposed part of the photofunctionalized implant attracted blood during surgery and was eventually covered with bone tissue, suggesting that the photofunctionalized implant functioned as a guide for bone regeneration [35]. Fast and strong osseointegration and the advantages in bone-healing mechanisms on photofunctionalized implants surface could support the establishment of osseointegration in the study patients with seriously compromised bone conditions. That is to say, photofunctionalization could be a powerful tool to establish secure osseointegration when the implant is placed with simultaneous bone augmentation.

Photofunctionalized implant in non-oral-cancer-related cases, but other regular and complex cases, showed long-term 100% success rate, which was not inferior to previous studies [25,26]. However, photofunctionalization could not increase success rate in oral-cancer-related cases in which not only bone and mucosa but also the salivary gland and neuromuscular system were compromised, indicating that pathophysiologically compromised oral condition is still a challenging aspect for dental implant treatment, particularly for long-term success.

## 5. Conclusions

Photofunctionalization of dental implants showed a promising result on regular and complex cases when the residual bone was not compromised due to oral cancer treatment. In cases of cancer-related sites, photofunctionalization could not show a promising effect to establish long-term success. Further clinical advancement and the development of additional techniques for photofunctionalization can be strong tools for the establishment of long-term implant stability, even in cancer-related pathophysiologically compromised jaws.

## Figures and Tables

**Table 1:** Case descriptions of the present study.

Groups	Regular	Complex	Cancer
Description	No site development or cancer-related issue	Simultaneous GBR, sinus lift, or fresh extraction sockets No cancer-related issue	Cancer-related resection with/without radiation
No. of implants placed	34 implants (4 implants in sleep)	8 implants with sinus lift 2 implants after sinus lift 8 implants with GBR 2 with absorbable membrane 6 with titanium mesh 3 implants in fresh extraction socket	4 implants in augmented bone after resection 4 implants in resection site with radiation history 7 implants in reconstruction site with radiation history
No. of implants analyzed	30 implants	21 implants	15 implants

GBR, guided bone regeneration.

**Table 2:** Implant profiles used in the present study.

Pt	Impl	Site	Qual	Qty	D	L	HP	R/C/Ca	Graft	Surgery	Times	Fo (mo)	Status	Cause
1	1	17	2	A	4	13	3	R				84	S	
	2	16	2	A	4	13	3	R				84	S	
	3	14	2	A	4	13	3	R				84	S	
	4	11	2	B	4	13	3	R				84	S	
	5	21	2	B	4	13	3	R				84	S	
	6	22	2	B	4	13	3	R				84	S	
	7	37	2	C	5	8.5	3	R				84	S	
	8	36	2	C	5	8.5	3	R				84	S	
	9	32	1	B	4	13	3	R				84	S	
	10	31	1	B	4	13	3	R				84	S	
	11	41	1	B	4	13	3	Ca				84	S	
	12	43	1	B	4	13	3	Ca				84	S	
	13	46	1	C	5	10	3	Ca				45	F	Periimplantitis
	14	47	1	C	5	10	3	Ca				45	F	Periimplantitis
2	15	45	2	B	4	13	3	C	Chin	GBR/Memb	1	84	S	
	16	46	2	B	4	13	3	C	Chin	GBR/Memb	1	84	S	
3	17	24	2	A	4	10	3	R				84	S	
4	18	16	1	E	4	13	6	C	PCBM	Sinus	1	84	S	
	19	14	1	E	4	13	6	C	PCBM	Sinus	1	84	S	
	20	13	1	D	4	15	6	C	PCBM	GBR/TiMesh	1	84	S	
	21	23	2	B	4	15	6	R				84	S	
	22	24	1	E	4	13	6	C	PCBM	Sinus	1	84	S	
	23	26	1	E	4	13	6	C	PCBM	Sinus	1	84	S	
	24	37	2	A	4	13	Sleep	R				-	W	Sleep
	25	47	2	A	4	13	Sleep	R				-	W	Sleep
5	26	16	2	B	4	10	3	R				84	S	
	27	17	2	B	4	10	3	R				84	S	
6	28	36	1	E	4	13	3	Ca	Fibular			5	F	Not integrate
	29	35	1	E	4	13	3	Ca	Fibular			44	W	Ca treatment
	30	33	1	E	4	13	3	Ca	Fibular			44	W	Ca treatment
	31	32	1	E	4	15	3	Ca	Fibular			44	W	Ca treatment
	32	31	1	E	4	15	3	Ca	Fibular			44	W	Ca treatment
	33	41	1	E	4	15	3	Ca	Fibular			44	W	Ca treatment
	34	43	1	E	4	15	3	Ca	Fibular			44	W	Ca treatment
7	35	16	3	B	4	13	6	C	PCBM	Sinus	1	84	S	
	36	12	3	EXT	4	13	6	C	PCBM	GBR/TiMesh	1	84	S	
	37	22	3	B	4	13	6	C	PCBM	GBR/TiMesh	1	84	S	
	38	24	3	B	4	13	6	C	PCBM	GBR/TiMesh	1	84	S	
	39	26	3	EXT	4	13	6	C	PCBM	Sinus	1	84	S	
	40	27	3	E	4	10	6	C	PCBM	Sinus	1	84	S	
	41	31	1→5	E→C	4	15	6	Ca	PCBM	GBR/TiMesh	2	84	F	Periimplantitis
	42	41	1→5	E→C	4	15	6	Ca	PCBM	GBR/TiMesh	2	84	F	Periimplantitis
	43	43	1→5	E→C	4	15	6	Ca	PCBM	GBR/TiMesh	2	84	F	Periimplantitis
	44	44	1→5	E→C	4	15	6	Ca	PCBM	GBR/TiMesh	2	84	F	Periimplantitis
	45	46	2	EXT	4	13	6	C				84	S	
	46	47	2	D	4	13	Sleep	R				-	W	Sleep
8	47	15	2	B	4	13	8	R				84	S	
	48	14	2	EXT	4	13	8	C	PCBM	GBR/TiMesh	1	84	S	
	49	13	2	B	4	13	3	R				84	S	
	50	11	2	B	4	13	3	R				84	S	
	51	24	2	EXT	4	13	8	C	PCBM	GBR/TiMesh	1	84	S	
	52	26	2	C	4	13	8	C	PCBM	Sinus	1	84	S	
	53	36	2	EXT	4	13	3	C				84	S	
	54	34	2	EXT	4	13	3	C				84	S	
	55	32	2	C	4	13	3	R				84	S	
	56	42	2	C	4	13	3	R				84	S	
	57	45	2	A	4	13	3	R				84	S	
	58	46	2	A	4	13	3	R				84	S	
9	59	26	1→4	E→A	5	10	6	C	PCBM	Sinus	2	84	S	
	60	27	1→4	E→A	5	10	6	C	PCBM	Sinus	2	84	S	
10	61	45	2	A	4.3	10	2	R				84	S	
	62	46	2	A	4.3	10	2	R				84	S	
11	63	15	1	B	3.5	10	3	R				84	S	
12	64	41	3	C	4	13	3	R				36	W	Ca treatment
13	65	15	3	A	4	10	3	R				84	S	
14	66	25	3	B	3.75	10	3	R				84	S	
	67	26	3	B	3.75	10	3	R				84	S	
15	68	14	3	B	4	13	3	R				34	W	Ca treatment
	69	17	3	B	4	8.5	Sleep	R				-	W	Sleep
16	70	14	2	B	4	13	3	R				84	S	

Notes: Impl, implant number; Site, FDI style, location to place implant; Qual, quality of the bone; QTY, quantity of the bone; D, diameter of the implant; L, length of the implant; HP, healing period (month) between implant placement and secondary surgery; slept, implant that did not proceed with secondary surgery. R/C/Ca, regular (R), complex (C), or cancer-related (Ca) sites; Graft, source of the graft bone; PCBM, particulate cancellous bone and marrow (if applicable); Surgery, procedure of the bone augmentation; Sinus, sinus elevation; GBR/Memb, guided bone regeneration with absorbable membrane; GBR/TiMesh, guided bone regeneration with titanium mesh (if applicable); Times, timing of the bone augmentation procedure, 1 = simultaneous bone augmentation and 2 = bone augmentation before implantation (staged approach) (if applicable); Fo, follow-up period (month); Status, implant condition after five-year follow-up; S, success; F, failure, implant that deviates success criteria (if applicable); W, withdrawn, implant that was not used prosthesis (sleep) or renounced to follow because of other disease treatment. Cause, reason of the failure of withdrawn (if applicable).

**Table 3:** Life table of photofunctionalized implants.

Time	Number of Implants			Cumulative Overall Success Rate (%)
	Total (Survival)	Failure (Lost)	Withdrawn	
Placement -Loading	70	0	4	100
Loading -Prosthesis	66	1 (1)	0	98.5
Prosthesis –1y (year)	65	0	0	98.5
1y–2y	65	0	0	98.5
2y–3y	65	4	0	92.3
3y–4y	65	2 (2)	8	87.9
4y–7y	55	0	0	87.9

**Table 4:** Success rates of implant in each category.

	7-Year Success Rate (%)
Overall	87.9
-Regular cases	100
-Complex cases (atrophied jaw or extraction socket)	100
-Cancer-related sites	22.2

**Table 5:** Implant stability quotient (ISQ) measurement score at implant placement and secondary surgery in overall, regular, complex, and cancer-related sites.

	ISQ1	ISQ2	ISQ Increase/Decrease
Overall	52.6	66.3	13.7
Regular	67.2	70.4	3.2
Complex	30.5	52.4	21.9
Cancer-related	62.1	58.6	−3.5

**Table 6:** Results of the multiple regression analysis.

Factor	Beta	Coefficients	SE	t	*p*
Age	−0.113	0.335	−0.063	0.412	0.683
Sex	−0.813	0.980	−0.114	0.746	0.459
Site1	−0.613	0.234	−0.326	2.138	0.042 *
Maxilla/Mandible					
Site2	0.182	0.333	0.074	3.483	0.021 *
Anterior/Posterior					
Bone quality	−2.190	0.192	−0.219	2.928	0.030 *
D2–4 or D1, 5					
Bone quantity	0.027	0.123	0.108	0.025	0.924
Diameter	0.711	1.023	0.061	0.387	0.299
Length	0.041	0.689	0.113	0.545	0.881
Healing period	0.926	1.032	−0.328	0.592	0.138
Resection	0.087	0.209	0.428	3.972	0.007 **
Radiation	1.876	1.992	0.133	1.433	0.092

* *p* < 0.05; ** *p* < 0.01.

## Abbreviations

BIC	bone–implant contact
Ca	cancer
GBR	guided bone regeneration
HU	Hounsfield Unit
ISQ	implant stability quotient
PCBM	particulate cancellous bone and marrow
RFA	resonance frequency analysis
UV	ultraviolet
